# Association of polymicrobial interactions with dental caries development and prevention

**DOI:** 10.3389/fmicb.2023.1162380

**Published:** 2023-05-18

**Authors:** Yimei Zhu, Ying Wang, Shuyang Zhang, Jiaxuan Li, Xin Li, Yuanyuan Ying, Jinna Yuan, Keda Chen, Shuli Deng, Qingjing Wang

**Affiliations:** ^1^Shulan International Medical College, Zhejiang Shuren University, Hangzhou, Zhejiang, China; ^2^Stomatology Hospital, School of Stomatology, Zhejiang University School of Medicine, Zhejiang Provincial Clinical Research Center for Oral Diseases, Key Laboratory of Oral Biomedical Research of Zhejiang Province, Cancer Center of Zhejiang University, Hangzhou, China

**Keywords:** cariogenic factors, microorganisms, quorum sensing, ecological perspectives, anti-caries, *Streptococcus* spp.

## Abstract

Dental caries is a common oral disease. In many cases, disruption of the ecological balance of the oral cavity can result in the occurrence of dental caries. There are many cariogenic microbiota and factors, and their identification allows us to take corresponding prevention and control measures. With the development of microbiology, the caries-causing bacteria have evolved from the traditional single *Streptococcus mutans* to the discovery of oral symbiotic bacteria. Thus it is necessary to systematically organized the association of polymicrobial interactions with dental caries development. In terms of ecology, caries occurs due to an ecological imbalance of the microbiota, caused by the growth and reproduction of cariogenic microbiota due to external factors or the disruption of homeostasis by one’s own factors. To reduce the occurrence of dental caries effectively, and considering the latest scientific viewpoints, caries may be viewed from the perspective of ecology, and preventive measures can be taken; hence, this article systematically summarizes the prevention and treatment of dental caries from the aspects of ecological perspectives, in particular the ecological biofilm formation, bacterial quorum sensing, the main cariogenic microbiota, and preventive measures.

## Introduction

**FIGURE 1:**
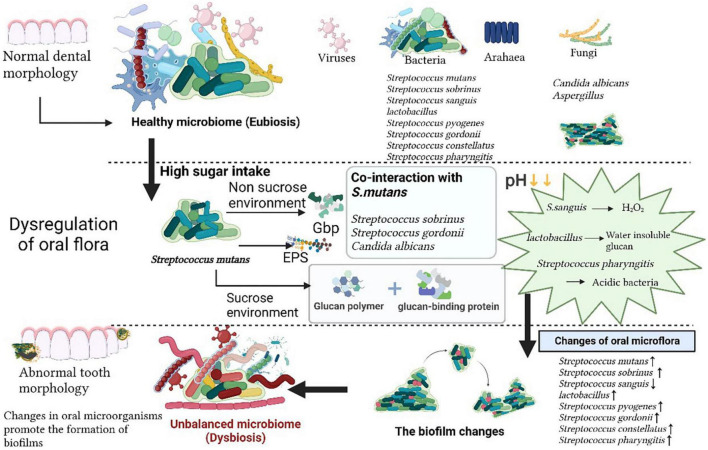
The oral microbiome. The normal homeostasis of oral microecology is characterized by a variety of microorganisms, such as viruses, bacteria, archaea, and fungi. Following high sugar intake, *Streptococcus mutans* secretes virulence factors via sucrose and non-sucrose pathways, which act in concert with other oral commensal flora, the pH in the oral environment decreases due to changes in the dominant flora, the oral microbial community is altered, the oral flora becomes dysbiosis and promotes biofilm formation, ultimately leading to the development of caries.

The oral cavity is a complex ecological environment and dental caries is formed by the combined action of bacteria-host-food-time. Caries is formed through a series of oral activities, such as biofilm formation and the bacterial metabolism of sugars within dental plaque to produce acid, followed by acid demineralizing tooth enamel. The “Ecological Plaque Hypothesis” (Marsh, 1994; Marsh and Devine, 2011; Rosier et al., 2014) is the mainstream theory of caries disease etiology, in which the occurrence of tooth decay involves a variety of microorganisms in dental plaque, the plaque between the tooth surface and the oral environment, and disruption of the ecological balance. Binding sites for cariogenic and other microorganisms are provided by the extracellular polysaccharide matrix, which is produced by cariogenic bacteria in biofilms. For example, the glucosyltransferases (Gtfs) of *S. mutans*, which is constituents of the membrane and also binds to microbial surfaces, provides the EPS in cariogenic biofilms (Koo et al., 2013), which provides a living environment for cariogenic bacteria. Excessive intake of sweets and carbohydrates is an important factor in the occurrence of caries, and external factors drive the oral microbiota from a balanced symbiosis with the host to a state of dysbiosis. Over time, the dominant microbiota is selected in the oral cavity, in which the acidogenic and aciduric microbiota plays a role in the demineralization of tooth enamel. The acidogenic microbiota that is enhancing demineralization is being selected for. The acid-producing and acid-resistant acid microbiota will prevail in an altered environment (Figure 1).

## Main cariogenic factors: microorganisms

Hundreds of microorganisms are present in the mouth. Indeed 16S ribosomal RNA (rRNA) probes confirmed the wide diversity of oral and caries microbiome species, the former is mainly composed of bacteria, fungus, virus, mycoplasma, protozoa, chlamydia, archaea and the latter mainly refers to *Streptococcus*, *Lactobacillus*, *Haemophilus*, and *Rothia* (Burcham et al., 2020; Figure 1). A variety of microorganisms interact and restrict each other, forming a balanced ecosystem. The oral microbiota, of which most are symbiotic bacteria, comprises beneficial and harmful microorganisms. The species composition of the gingival microbiota changed from the symbiotic patch biofilm community to the ecologically disregulated community dominated by acidic and aciduric species (Dashper et al., 2019). When the over proliferation or absence of a certain microorganism breaks this balance, various diseases can occur. The oral disease, dental caries, is not caused by the introduction of exogenous pathogens into the oral environment, but by the breakdown or disruption of the balance in the structure of the microbial community existing in a normal healthy state (Valm, 2019). The main cariogenic factors are described as follows: traditional cariogenic bacteria which recognized as caries-causing bacteria strains early, symbiotic cariogenic bacteria which symbiosis with caries-causing bacteria enhance caries-causing effects, and cariogenic fungi which plays a synergistic role (Table 1).

**TABLE 1:** Caries-causing factors.

Perspective			Representatives	Molecular mechanism	Specific regulatory factors	References
Ecological perspectives	The caries ecological hypothesis		Glucan production	Gtf-derived dextran helps to accumulate bacterial cells	Gtf	Falsetta et al., 2014
			Bacterial-fungal sugar metabolism	*Candida albicans* induces the expression of *S. mutans* virulence genes and enhances plaque Gtf activity	*Candida albicans*, *S. mutans*	
			Colonization of acidic dominant flora	The microenvironment in the mouth is changed, acidity is increased, acid-resistant bacteria are colonized, and the environment is driven to an acidic state	Acidic bacteria	
	Genetic factors		Low caries gene loci	Mutations in the genes of organic matrix molecules can lead to the production or absence of abnormal proteins, resulting in mineralization defects, affecting bacterial adhesion or resistance to acidic environments, increasing tooth sensitivity and thus leading to dental caries	5q13.3, 14q11.2, Xq27.1	Opal et al., 2015
			High caries gene loci		13q31.1, 14q24.3	
			Immune response	Modification of caries susceptibility through immune response	Gene Fragments *HLA-DR4*, *DR3*, *TNFα117*	Vieira, 2012
					Major histocompatibility complexes	
			SCPP gene	*SPP1* encodes a protein that is expressed in tissues and is involved in mineralized tissue remodeling (Hasan et al., 2022)	*SPP1*, *MEPE*, *IBSP*, *DMP1*, *DSPP*	Eckert et al., 2017
Microbiological perspective	Bacterium	Traditional cariogenic bacteria	*Streptococcus mutans*	Colonizes the tooth surface and participates in interspecies competition within the plaque biofilm, which in turn leads to biofilm alteration	Gbp, EPS	
		Symbiotic cariogenic bacteria	*Streptococcus sobrinus*	Forming a cariogenic microenvironment with other streptococci	ComRS systems	Underhill et al., 2018
			*Streptococcus sanguinis*	Hydrogen peroxide produced by blood streptococci provides an acidic environment for cariogenesis	Hydrogen peroxide	
		Other bacteria	*Lactobacillus*	Promotes bacterial attachment to tooth surfaces, promotes plaque build-up and limits organic acids	water-insoluble polysaccharide	
			*Streptococcus pyogenes*	Formation of microenvironment-dependent host-pathogen protein complexes	GlycerAldehyde 3-Phosphate DeHydrogenase (GADPH)	Chaudhary et al., 2021
			*Streptococcus Gordonii*	Produces alkali, regulates pH fluctuations in the mouth, adheres to dental plaque, and works in conjunction with other cariogenic bacteria	ATP-binding cassette (ABC)-type transporters	
			*Streptococcus constellatus*	In case of septic infection of the tooth		
			*Streptococcus pharyngitis*	Acidic bacteria colonize in an acidic environment		
	Cariogenic fungi		*Candida albicans*	Bacterial-fungal co-interaction		

### Streptococcus mutans

*S. mutans* is considered the main cariogenic bacteria in the oral cavity. There are obvious differences between the cariogenic microbiota and the salivary microbiota of different people (caries-active and caries-free), and the cariogenic microbiota is characterized by the presence of a relatively high abundance of *S. mutans*, *Bifidobacterium bifidum* and so on (Hurley et al., 2019). *S. mutans* colonizes tooth surfaces via sucrose independent and sucrose dependent mechanisms. *S. mutans* exposes non-enzymatic glucose-binding proteins (Gbps) on its cell surface, and a majority of glucosyltransferase catalyzes the glucosyl transfer of sucrose to glucan chains (Devulapalle et al., 1997). *S. mutans* produces four glucan-binding polymerases (Gbp) that play a vital role in bacterial adhesion and adsorption to tooth enamel, providing a niche for microbial colonization and an insoluble matrix for dental plaque, which is the material basis for caries (Bowen and Koo, 2011). The amino acid sequences of the four types of Gbp(A-D) have limited homology, and GbpA and GbpB are derived from dextran matrix adhesion, while GbpC is derived from a mutant lacking dextran-dependent aggregation (DDAG) (Sato et al., 2002; Lynch et al., 2013), which is expressed only under stress conditions when it comes into contact with dextran-induced aggregation, GbpD is a glucan binding protein identified in the complete annotated sequence of the *S. mutans* genome (Shah and Russell, 2004). Interspecific competition within the various oral microorganisms contributes to the cariogenicity of *Streptococcus* via biofilm alteration (Lynch et al., 2013). Glycosyltransferase (GTF) enzymes gtfB, gtfC, and gtfD produce water-insoluble glucan polymers in the presence of sucrose, which adhere to and accumulate in biofilms (Lemos et al., 2005). GtfB synthesizes water-insoluble glucans rich in α(1-3)-linkages, while GtfC produces a mixture of soluble glucans rich in α(1-6)-linkages and insoluble glucans, and GtfD makes primarily soluble glucans (often called dextran) (Lemos et al., 2019). Glucan encourages the accumulation of cariogenic *Streptococci* on the teeth, increasing the pathogenicity of dental plaque. Extracellular polysaccharide (EPS) is also one of the main cariogenic virulence factors of *S. mutans*. Ecological studies have also shown that *S. mutans* is more capable of surviving and thriving in the low pH conditions produced by sugar metabolism by inhibiting the growth of other oral bacteria, thereby contributing to the survival of *Streptococcus* (Bradshaw and Lynch, 2013). Low pH can also trigger demineralization of tooth enamel and thus lead to the formation of dental caries. *S. mutans* promotes the development of caries or provides a cariogenic material base in many ways. Thus, *S. mutans* clearly provides the material basis for caries.

### Streptococcus sobrinus

The *S. mutans* group contains not only the traditional cariogenic bacteria *S. mutans* but also cariogenic bacteria *Streptococcus sobrinus*. *S. sobrinus* has been described as associated with caries for decades. The symbiosis of *S. sobrinus* with other cariogenic bacteria is more likely to facilitate the development of caries. Compared with children without dental caries, children with active caries contained more specific microorganisms in their mouths, and the isolation rates of *S. mutans*, *S. sobrinus*, and *Candida albicans* were 66, 11, and 18% (Fragkou et al., 2016). Although *S. sobrinus* lacks the natural competence pathways used by other streptococci to regulate growth, virulence, and population sensing, a new class of ComRS-competent systems which can control the process of DNA transformation, that is adaptive response contributing to genome plasticity and virulence, widely present in most *S. sobrinus* strains (Ledesma-Garcia et al., 2020). Two ComR genes and an XIP sequence without any aromatic amino acids are unique features of the ComRS system, making it very similar to the cariogenic *S. salivarius* group pathway. *S. sobrinus* can co-exist with *S. salivarius* and *S. thermophilus* to form a cariogenic microenvironment (Li J. W. et al., 2021). With the improvement of detection means and detection sensitivity, the detection rate of *S. sobrinus* in the oral cavity has improved significantly. SB-20M medium could be used for the morphological identification of *S. mutans* and *S. sobrinus*, and the checkerboard DNA-DNA hybridization (CDDH) assay could be used to evaluate oral cariogenic bacteria such as *S. mutans* (Saravia et al., 2020) and *S. sobrinus* in the oral cavity of patients with fixed orthodontic appliances. The co-occurrence of *S. mutans* and *S. sobrinus* can worsen caries (Islam et al., 2007).

### Streptococcus sanguinis

*Streptococcus sanguinis* was found to be the bacterium with the highest relative abundance in the dentition connective tissue dysplasia (CTD) oral biofilm microbiome using next-generation sequencing (HOMINGS, Human Oral Microbe Identification using Next Generation Sequencing) technology (Harris-Ricardo et al., 2019). As symbiotic members of the early colonizers in dental biofilms, the effect of the relative abundance of *S.sanguinis* in oral biofilms is reflected in the overall functional expression profile of dental plaque microorganisms (Kreth et al., 2017), and a decrease in the relative abundance of blood streptococci is one of the hallmarks of caries development. Therefore, monitoring the relative abundance of streptococci in the blood is an effective way to predict dental caries. Hydrogen peroxide produced by *S. sanguinis* might alter the pH in the oral cavity, leading to an increase or decrease in cariogenic bacteria, thereby influencing the progression of caries (Vaknin et al., 2020). When pH is reduced, acid resistant dominant bacteria such as *S.mutans*, *Lactobacillus* and *S.anginosus* are more likely to grow and reproduce, while the proliferation of *S.sanguis* decreases due to its unsuitable growth characteristics. There are also other environmental factors that can affect microbial population fluctuations. The number of *S. sanguinis* in the mouths in children without caries is higher than that of *S. mutans*, while the abundance of *S. sanguinis* in early severe caries of children is reduced and is lower than that of *S. mutans*. The interaction between *S. sanguinis* and *S. mutans* leads to an increased incidence of dental caries in children. A study indicated that there were differences in the bacterial content of high and low caries biofilms, with *S. sanguinis* being more abundant in individuals with high caries biofilms (AlEraky et al., 2021). *S. sanguinis* not only affects the oral microenvironment, but also is the cause of infective endocarditis; therefore, its detection in the oral cavity is significantly more important.

### Cariogenic fungi

Among the cariogenic microorganisms, certain fungi, such as *Candida albicans*, can form a symbiosis with cariogenic bacteria and are more likely to change the sensitivity of teeth to caries formation. As a symbiotic fungus, *C. albicans* evades host defenses and colonizes new environments by penetrating tissues, where it is exposed to new adherence receptors and responds by expressing alternative adhesins (Cannon and Chaffin, 1999; Naglik et al., 2003; Hernández-Solís et al., 2014). Large numbers of *C. albicans* and *S. mutans* can be detected in the biofilm of early childhood caries. Research has shown that most strains have a genetic correlation, and the source of *C. albicans* in children comes from their mothers, with carriage rates of *C. albicans* in saliva and dental plaque of children with severe early childhood caries and their mothers being more than 80% (Xiao et al., 2016). *C. albicans* induces the expression of *S. mutans* virulence genes and enhances plaque GTF activity. Candida-derived β1,3-glucan provides favorable conditions for EPS formation, while GtfB binding and active sites are provided by fungal mannan and β-glucan. Compared with *S. mutans* or *C. albicans* alone, co-infection with symbiotic bacteria and the fungus enhances the toxicity of the biofilm (Falsetta et al., 2014), leading to a marked increase in the possibility of dental caries.

### Other bacteria

The occurrence of dental caries is also closely related to *Lactobacillus*, *Streptococcus pyogenes*, *Streptococcus gordonii*, *Streptococcus constellatus, Streptococcus mirabilis*, *Streptococcus salivarius*, and *Streptococcus pharyngitis*. Unlike *S. mutans*, *Lactobacillus* is a floating, opportunistic settler in the oral cavity and can only colonize certain restricted ecological niches (Caufield et al., 2015). The water-insoluble polysaccharide produced by *Lactobacillus* promotes bacterial adhesion to the tooth surface, facilitating plaque accumulation and restricting organic acids to alter the microenvironment (Zheng et al., 2019). Decayed, missing, or filled tooth surfaces (DMFS) or decayed/missing/filled teeth (DMFT) scores are accepted disease parameters in dental caries clinical trials (Ferraro and Vieira, 2010; Mendes et al., 2010). There is a correlation between DMFT and the prevalence of *Lactobacillus* in the oral cavity, indicating that *Lactobacillus* counts are reliable in determining caries activity (Botha et al., 2001). Species, such as *S. Sanguinis*, *Streptococcus oralis*, *Streptococcus mielii*, *S. gordonii*, and *S. anginosus*, are major colonizing bacteria on the tooth surface. As human oral symbiotic bacteria, they are closely related to oral microecological homeostasis. *S. gordonii* and *S. oralis* microbiota produce large amounts of alkali, which regulate pH fluctuations in the oral cavity. They can also adhere to dental plaque via specific adhesin-receptor interactions (Choo et al., 2021), co-aggregating with other cariogenic bacteria and playing a common role. *S. mutans* forms microenvironment-dependent host-pathogen protein complexes that counter immune surveillance of the mucosa and express multiple virulence factors on its surface to localize and initiate human infections (Chowdhury et al., 2021). *S. constellatus*, which can cause tooth decay and odontogenic infections, is also a commensal organism that is often present in the oral microbiota. The acidity induced by *S. pharyngeus* might increase the possibility of colonization in an acidic environment, leading to the development of caries and even bacterial systemic infection (Sasaki et al., 2018). *S. alivarius* can indirectly promote oral halitosis by deglycosylating salivary glycoproteins, making the protein core available for further degradation by gram-negative bacteria. The extensive production of extracellular aggregates of *S. salivarius* and *S. anginosus*, in combination with *S. mutans*, in the sucrose environment, which can be considered an indicator of caries activity in patients with active tooth decay, promotes bacterial attachment and biofilm formation (Chen et al., 2020).

## Mechanism of the quorum sensing in the pathogenesis of dental caries

Interbacterial communication systems allow bacteria to disseminate information about their environment, metabolism, and other survival information via extracellular signal transmission, which induces a series of responses in the body.

In gram-positive bacteria, quorum sensing (QS) is coordinated by peptide pheromones, which act as extracellular signaling molecules to sense changes in gene expression and ultimately activate a coordinated population response (Lyon and Novick, 2004; Shanker and Federle, 2017). Two peptide pheromones: autoinducer-2 (AI-2) (Niazy, 2021) and competence-stimulating peptide (CSP) (Bikash and Tal-Gan, 2020), are used by most oral bacteria to communicate for biofilm production and play an important role in the development of biofilms.

### QS system of *Streptococcus mutans*

Scientists studied the relationship between bacterial QS and caries-causation as well as the contribution of *S. mutans* to the process of dental plaque formation. *S. mutans* possesses a short hydrophobic peptide (SHP)/Rgg quorum sensing system that regulates a specific biosynthetic manipulator with the radical-SAM (S-adenosyl-l-methionine) (RaS) enzyme and produces ribosomally synthesized and post-translationally modified peptides (Rued et al., 2021). The growth pattern of dental plaque is regulated by the density-dependent QS system of *S. mutans. S. mutans* coordinates genetic transformation through two peptide pheromones CSP and comX inducing peptide (XIP). This QS system plays key role in adapting to the harsh environment conditions of bacteria, including oral biofilm formation, genetic capacity, acid production, bacterial virulence activity, and EPS production (Zhang et al., 2009; Kaur et al., 2015; Acet et al., 2021).

#### CSP

One of the main virulence factors of CSP is peptide antibiotics, which can facilitate microorganisms to compete for limited nutrients in the environment. CSP is sensed by the ComCDE system and induces competence indirectly. Competence is regulated by an intercellular communication system based on secreted peptide signaling molecules (Bikash and Tal-Gan, 2020). 21-CSP is secreted by *S. mutans* and is processed by the membrane-bound protease SepM to active 18-CSP, which then binds to the ComD receptor, contributing to biofilm formation in the oral cavity (Hossain and Biswas, 2012; Bikash et al., 2018). The growth pattern of dental plaque related to *S. mutans* is regulated by the density-dependent QS system of *S. mutans*, which consists mainly of CSP and the ComD/ComE two-component signal transduction system (Senadheera and Cvitkovitch, 2008).

#### ComX

**FIGURE 2:**
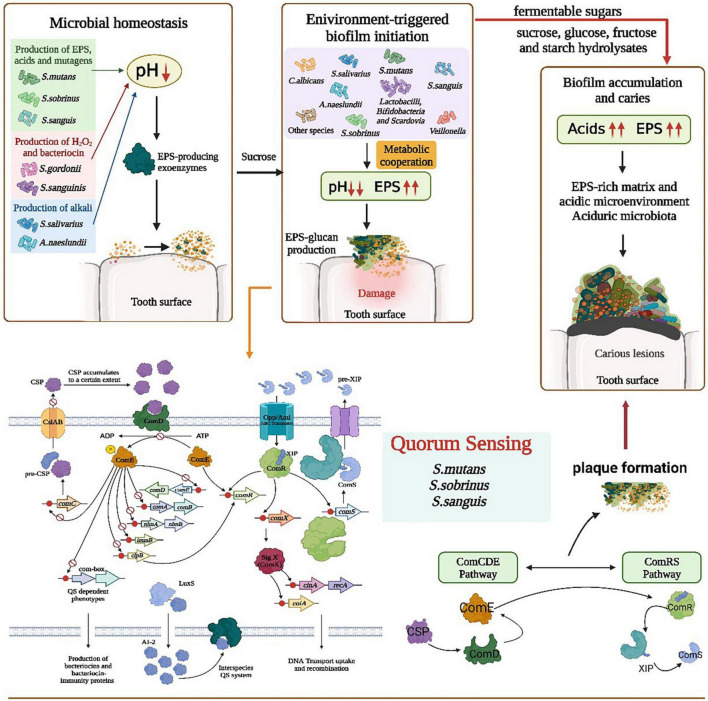
Role of polymicrobial interactions in the process of caries occurrence. The ecological balance in the mouth is broken because of the excessive reproduction or loss of certain bacteria or fungi, probably due to the production of EPS by microorganisms such as *S. smutans*, H_2_O_2_ by *S. gordonii*, and alkaline substances by *A. naeslundii* that disrupt the environmental homeostasis in the oral cavity, followed by the enrichment of various bacteria on the tooth surface. *S. mutans/S. sobrinus/S. sanguis* quorum sensing through the metabolic synergism of ComCDE and ComRS pathways resulted in lower pH, increased EPS, production of dextran attached to the tooth surface. Also CSP, ComD/ComE dual component and LuxS/AI-2 QS system in the quorum sensing contribute to plaque formation and tooth damage. With the intake of fermented sugars, the acid in the mouth increases, EPS increases, and in the acidic environment and EPS-enriched environment, biofilm forms on the tooth surface, finally resulting in dental caries.

The virulence and cariogenicity of *S. mutans* are also attenuated when two signaling systems, ComCDE and HK/RR11, are inactivated simultaneously (Li et al., 2008). A study showed that the expression of *ComD* and *ComX* was significantly upregulated in the persistent strain of *S. mutans* compared with that in the seeds (*S. mutans* cells), and persistence was significantly enhanced at an early stage compared with untreated seed biofilms, with the expression of *vicR* being particularly pronounced (Lu et al., 2019). *Mutants* defective in ComC, -D, or -E gene expression (encoding a population-sensing system essential for cell density-dependent induction of heritability) have reduced the log phase acid resistance response (ATR) in *S. mutans* biofilm cells. A synthetic CSP was added to comC mutants to restore the ATR (Li et al., 2001; Figure 2).

#### LuxS/AI-2 QS system

**FIGURE 3:**
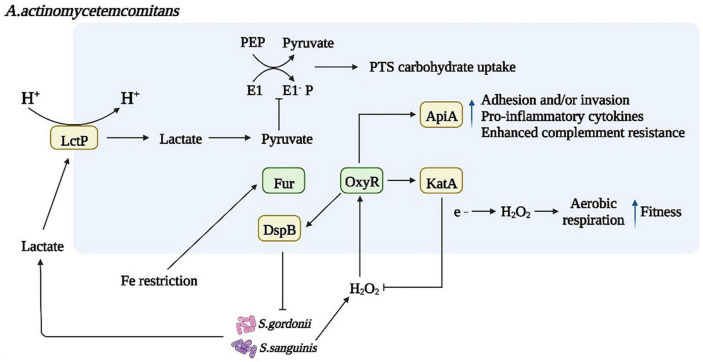
Microbial interactions affect bacterial metabolism and biofilm formation. Dysregulated inflammation contributes to an environment rich in oxidative stress, and *S. gordonii* and *S. sanguinis* activate the oxygen resistance transcriptional regulator (oxyR), which regulates the expression of Omp100 and cytoplasmic catalase (KatA), degrading H_2_O_2_ and protecting bacteria from oxidative damage. As a result, increasing oxygen availability enables the bacteria to transition from fermentation to respiratory metabolism. *A. act* can use *S. gordonii* metabolite H_2_O_2_ as a signaling molecule to induce the expression of *katA* and *apiA*, the products of which contribute to resistance to host innate disease immunity. Another metabolite of *S. gordonii* and *S. sanguinis*, lactate, is produced in large quantities under the action of lactate permease in an acidic environment, promoting increased pyruvate subject to pyruvate signaling. Sugar Phosphotransferase System (PTS) in bacterial phosphoenolpyruvate mediates carbohydrate uptake and phosphorylation inducing biofilm formation through the direct and indirect action of the PTS.

Another *S. mutans* QS mechanism uses AI-2, a byproduct of LuxS-mediated methyl metabolism. The LuxS/AI-2 QS population sensing system inhibits the formation of mixed species biofilms of *S. mutans* and dental surface colonizing bacteria such as *S. gordonii* (Wang et al., 2017). The LuxS/AI-2 QS system is involved in biofilm formation, acid tolerance, and acid production in *S. mutans* and the expression of related genes. AI-2 has an important role in *S. mutans* biofilm formation and can upregulate cariogenic genes (*spaP*, *fruA*, *gtfB*, *gtfC*, and *gtfD*). LuxS mutations impair biofilm formation, acid resistance, and acid production, and this damage is the result of AI-2-mediated QS. In addition, intact QS regulators of *S. mutans* can be induced by unrelated oral pathogens, such as *Aggregatibacter actinomycetemcomitans* (*A. act*) (Figure 3). *A. act* can use the metabolite H_2_O_2_ of *S. gordonii* as a signaling molecule to induce the expression of cytoplasmic catalase (KatA) and complement resistance protein ApiA (Ramsey and Whiteley, 2009; Jeckelmann and Erni, 2019). In the presence of *A. act.* and downregulated oxidative stress-related genes, *S. mutans* upregulated the intact QS regulon (transformants and mutagens) (Szafrański et al., 2017a). Another metabolite of *S. gordonii* and *S. sanguinis*, lactate, promotes increased pyruvate to mediate carbohydrate uptake and thus induce biofilm formation (Waseem et al., 2019).

#### VicRK system

The VicRK system, which is one of the regulatory network two-component signal transduction systems (TCS) in *S. mutans*, is the key regulator of acidification and biofilm formation (Lei et al., 2019). Genes in the VicRK pathway are upregulated before chronic fast regeneration of *S. mutans*, which enhances the regeneration of *S. mutans* and increases the recurrence of dental caries (Lu et al., 2019). TreR in *S. mutans* can act as a transcription factor for the TreR operon encoding a trehalose sensing activator. Trehalose is a disaccharide commonly found in human food, and the absence of TreR inhibits *S.mutans’* ability to prevent the development of competing species such as *S.gordonii* and *Lactococcus Lactis* (Baker et al., 2018). It was found that methanolic extracts of *Camellia sinensis* and *Psidium* spp. could exert anti-colony sensing ability against oral pathogens, while cranberry extracts affected the acid production and metabolic ability of *S. mutans*; therefore, substances in plant extracts could inhibit QS genes and QS control factors, thus interfering with biofilm accumulation. *S. mutans* density sensing is closely related to the occurrence of caries. QS and the upregulation of two-component system VicRK pathway genes during the lag time before persistent rapid regeneration of *S. mutans* promotes the regeneration of *S. mutans* and increases caries recurrence (Lu et al., 2019). AHL is a QS signal produced by gram-negative bacteria, and Muras et al. demonstrated the role of AHL-mediated QS in plaque formation and its possible involvement in the ecology of ecological disorders (Muras et al., 2020). For example, the broad-spectrum AHL-lactonase, Aii20J, significantly inhibits oral biofilm formation in different *in vitro* biofilm models and can cause important changes in bacterial composition.

Density sensing of *S. mutans* is strongly associated with caries ecology. Schramm et al. found that MTAN, an analog of methylthioadenosine nuclease, can interrupt the AI-2 population sensing system by providing an intermediate analog that interferes with the biochemical synthesis of the AI-2 protein, thereby reducing or even preventing biofilm synthesis (Schramm, 2007). A recent study by Ryu et al. found that D-galactose inhibits biofilm formation in *S mutans* (Ryu et al., 2020).

## Ecological perspectives

### The caries ecological hypothesis

People’s understanding of “one pathogen, one disease” of caries has been changed to an imbalance of oral microorganisms. With frequent carbohydrate consumption or reduced saliva, “positive feedback loops” within the microbial community can from, for instance, acidification caused by residual fermentation of carbohydrates in the mouth is regulated by a series of positive feedbacks or a chain of self-reinforcing events that selectively promote caries-causing or pathogenic microbiota. disrupting resilience and provoking changes in the ecosystem function and structure (a microbial “regime shift”), thus promoting ecological disorders and oral diseases (Rosier et al., 2018). An increase or decrease in the number of certain microbiota in the composition of the microbiota might cause dysbiosis, which is associated with certain oral and systemic diseases (Yamashita and Takeshita, 2017). Cariogenic bacteria-mediated and fermentable carbohydrate-driven dynamic changes in microbial communities, and the presence of genes for specific metabolic pathways, contribute to the occurrence and progression of dental caries.

According to the extended caries ecology hypothesis, the development of caries can be divided into three reversible processes – the dynamic stabilization stage, the acid production stage, and the aciduria stage (Takahashi and Nyvad, 2011). Under normal conditions, the pH in the biofilm does not fluctuate significantly because of the metabolic reactions of the microbial community remaining in a state of equilibrium (Nyvad and Takahashi, 2020); however, small changes in the local oral environment can have a significant impact on oral bacterial competitiveness. The occlusal surface is conducive to bacterial adhesion. In addition, the sulcus-fosse system, the relatively long eruption period, the decrease of oral mechanical function, and the metabolic activity in the mouth can become unbalanced. These occlusal surfaces, as mechanical protective sites, can promote the accumulation and maturation of biofilms, which can also develop into cariogenic biofilms (Carvalho et al., 2016; Liu et al., 2020), Gtf-derived dextran helps to accumulate bacterial cells (Falsetta et al., 2014). However, because of the frequent intake of carbohydrates, the oral cavity microorganisms will shift to a more acidic plaque microbiota (Georgios et al., 2015), which also enhances the acidity and acidity of non-proteobacteria. Enhanced bacterial-fungal sugar metabolism and acid production is associated with caries onset when *Candida albicans* is symbiotic with *S. mutans* in a sucrose-rich environment (Ellepola et al., 2019). Dietary carbohydrate-induced enrichment of plaque microbiota members, such as *S. mutans* and *Lactobacillus*, results in lower pH and increases the caries-causing potential of plaque. In addition to *S. mutans* and *Lactobacillus*, other bacteria capable of acidogenesis at low pH, such as *Actinomyces spp.* and *Bifidobacterium*, have caries-causing traits, including acidogenicity and acidity (Lamont et al., 2018), which might also contribute to the high pH reduction potential exhibited by many plaques. More acidic bacteria then select dominant microbiota through temporary acid damage and acid inhibition of growth (Takahashi and Nyvad, 2011), and the weak organic acids produced as by-products of fermentation carbohydrate metabolism contributes to the demineralization of tooth enamel (Inchingolo et al., 2022). Carboxyl groups of acidic proline-rich polypeptides promote the anchoring of oral microbial communities in cell membranes and enhance the colonization of tooth surfaces (Laputková et al., 2018). Although dental caries is considered an endogenous infection, exogenous environmental factors such as carbohydrate consumption in the diet, saliva flow, component dysfunction, and poor oral hygiene, regulate not only the composition of the oral bacteria, but also their activity and function, as well as influencing the process of caries development (Sánchez et al., 2022). *S. mutans* and other acidic bacteria can increase and promote lesion development by maintaining an environment characterized by a “net mineral loss” (acid phase) (Table 1).

### Genetic factors

Susceptibility to dental caries is linked to genetic factors, which might also lead to changes in the microbial ecology of the disease. Genetic factors have an influence on caries susceptibility and can independently mediate sucrose sweetness preference. Genetic factors account for up to 65% of the individual variation in caries experience (Shaffer et al., 2015).

Enamel is controlled by the interaction of many organic matrix molecules, and mutations in the genes encoding these organic matrix molecules can lead to the production or absence of abnormal proteins, resulting in mineralization defects, affecting bacterial adhesion or resistance to acidic environments, increasing tooth sensitivity, and thus leading to dental caries (Opal et al., 2015). The susceptibility to dental caries is related to some genetic loci. A potential association between a low caries level and loci 5q13.3, 14q11.2, and Xq27.1 was found, while a high caries level was associated with loci 13q31.1 and 14q24.3 (Vieira, 2012). Genetic variation in genes affects more mild phenotypic variation, such as the carbonic anhydrase (CA) VI gene (rs17032907) genetic variant and a haplotype of CA VI (ACA), and thus affects susceptibility to dental caries in non-syndromic cases (Bretz et al., 2005; Li et al., 2015). Deficiency of transcriptional repressor GATA binding 1 (TRPS1), a transcription factor involved in tooth development and mineralization of bone and tooth tissue, impairs dentin formation and enamel microhardness, thereby increasing the likelihood of mineral loss under acidic conditions (Socorro et al., 2022). For the cariogenic bacteria *S. mutans*, most of the shared gene order and configuration are preserved, but there are still dozens of genes that undergo genetic recombination, leading to differences in pathogenicity, and the alteration of virulence-related genes largely changes the cariogenic ability (Bedoya-Correa et al., 2019).

One aspect of the genetic effect is an altered immune response, in which polymorphisms in the major histocompatibility complex (MHC) might lead to immune responses against the level of oral colonization and might influence an individual’s susceptibility to dental caries (Opal et al., 2015). There is a potential link between immune response and *HLA-DR4* (encoding the major histocompatibility complex, class II, DR beta 4), *HLA-DR3, TNFα* (encoding tumor necrosis factor-alpha), and other genes in the oral cavity, and dental caries: a positive *HLA-DR4* allele might increase the risk of early childhood caries (Opal et al., 2015). Genetic and immunological differences between hosts might also be important risk factors for dental caries. *S. mutans* can stimulate the production of pro-inflammatory cytokines, the levels of which are positively correlated with salivary interleukin (IL)-1β concentrations and correlate negatively with salivary IL-1 receptor antagonist concentrations (Hu et al., 2019). Both distal-less homeobox 3 (DLX3), which plays a role in odontogenesis, and DLX4, which is highly expressed in human dental pulp cells (DPC), might play an important role in the process of dental caries by regulating tooth development (Alotaibi et al., 2021). Actinin Alpha 2 (ACTN2) (and other actin and cytoskeleton-related proteins) might be involved in tissue-forming cells during tooth enamel formation and play a role in caries susceptibility (Shaffer et al., 2011; Stanley et al., 2014). Dentin extracellular matrix secretory calcium-binding phosphoprotein (SCPP) genes [i.e., *SPP1* (encoding secreted phosphoprotein 1), *MEPE* (encoding matrix extracellular phosphoglycoprotein), *IBSP* (encoding integrin binding sialoprotein), *DMP1* (encoding dentin matrix acidic phosphoprotein 1), and *DSPP* (encoding Dentin Sialophosphoprotein)] are expressed in many tissues and are involved in mineralized tissue remodeling, which among various other functions, are key genes involved in biomineralization (Eckert et al., 2017). The occurrence of dental caries is also associated with parentage, and the mothers of children with severe dental caries associated with *C. albicans* also contain high concentrations of the same species. A large number of microbial species in dental plaque biofilms are closely related to the host saliva (Phattarataratip et al., 2011). Saliva contributes to biofilm proliferation while colostrum plays the opposite role, and 3’-sialolactose significantly reduces the formation of biofilms (Faria et al., 2022). Thus, the occurrence of dental caries and genetic factors are inseparable (Table 1).

## Development of preventive measures

Most studies have concluded that nanoparticles have advantages in restoring eubiosis or homeostasis. Moreover, nanobacterial agents, as new antibacterial supplements to antibiotics, can fill the deficiency of antibiotics that often fail in dentistry because of their broad-spectrum antibacterial properties and good stability (Besinis et al., 2014). In addition to inorganic nanoantimicrobial agents, organic nanoantimicrobial agents, such as chitosan nanoparticles and quaternary polyethyleneimine nanoparticles, have also been used in dentistry. Chitosan nanoparticles have a promising application in different fields of dentistry as a rinsing agent for root canal treatment with plaque removal and inhibition of bacterial reattachment (Nehra et al., 2018). Quaternary polyethyleneimine nanoparticles exert good antibacterial properties against pathogenic bacteria in the oral cavity by binding to proteins in bacterial cell walls or lipid-like layers on cell membranes, stopping bacterial cells from exchanging substances, resulting in cell death (Makvandi et al., 2018).

With the advancement of technology, many detection techniques can also indirectly play a role in the prevention and treatment of caries. The use of X-ray photography for the visual or visual-tactile (spherical probe) assessment of dental caries is generally the clinical standard, and radiography is recommended as an additional method in routine clinical practice (Braga et al., 2010). Quantitative light-induced fluorescence (QLF) for caries assessment, a method originally developed to detect early caries lesions, is limited to the measurement of demineralization by quantifying green fluorescence loss as an indirect measure of demineralization. SoproLife is a camera that illuminates the tooth surface within an excited radiation band of light to induce fluorescence. It can detect three types of enamel caries lesions by combining the advantages of a laser fluorescence device and oral endoscopy early in the disease process (Zeitouny et al., 2020). The incidence of dental caries can be assessed by monitoring bacterial abundance, protein properties and concentrations, and buffering capacity. Currently for non-cavitary lesions, the most convenient way to predict the tendency of lesions is to assess enamel roughness. Combined with the micropore models, the degree of dental caries damage can be judged by observing the caries activity of the saliva donor, the enamel caries-like lesions, and caries-causing microscopic biofilms produced under the inoculation conditions (Viana et al., 2021). The Nyvad and the International Caries Detection and Assessment System-II (ICDAS-II) visual systems, Nyvad uses a visual-tactile caries classification system to detect the activity and severity of caries lesions, while ICDAS records detailed information on the severity of caries through highly time-consuming measurements, can estimate the depth of deciduous teeth caries, and can accurately reveal the severity of caries to allow more rapid selection of treatment plans (Campus et al., 2019).

### Probiotics prevent dental caries by improving oral microecology

At present, a radical cure for caries has not been found. Majority of the caries therapy methods are outdated (e.g., penicillin, quinolones, aminoglycosides) or unviable other methods. Therefore, preventive measures can be taken according to the mechanisms of various cariogenic factors to reduce the occurrence of caries. From the perspective of dental caries ecology, probiotics can be used in combination with traditional antimicrobial peptides and antimicrobial small molecules for prevention. Numerous studies have confirmed that probiotics not only maintain the balance of intestinal microbiota, but also have beneficial effects on our skin, genitourinary tract, and oral cavity (Roudsari et al., 2015; Sihra et al., 2018; Zhang et al., 2018).

Studies have shown that probiotic-containing gum and xylitol can be used as an alternative to fluoride supplements to prevent dental caries in children (Caglar et al., 2007), and daily consumption of ice cream and curds containing the probiotic *Bifidobacterium* reduces salivary levels of *S. mutans*. Probiotics help to stimulate health-promoting microbiota and inhibit pathological colonization and disease, which can prevent dental caries by inhibiting the growth of cariogenic bacteria and symbiotic microbes in the oral cavity by buffering the pH of the saliva, inhibiting the production of bacteriocins and enzymes (glucanase, protease, and urease), and competing for tooth surface adhesion and colonization (Manmontri et al., 2020; Sivamaruthi et al., 2020). After the addition of probiotics, lactic acid bacteria strongly inhibit the growth of oral *Streptococcus*, and *Lactobacillus* might inhibit the formation of oral biofilms by reducing glucan production and the antibacterial activity of *S. mutans* (Lee and Kim, 2014). The use of probiotics can reduce *S. mutans* CFU counts (Seminario-Amez et al., 2017), which might prevent the occurrence of dental caries. In addition to conventional physical and chemical treatments, a new alternative to caries management is treatment using genetically modified “effector strains” of cariogenic bacteria. They may act as probiotics, helping to prevent colonization by cariogenic bacteria by producing ammonia, and also helping to maintain internal pH homeostasis. *Bifidobacterium* and *Lactobacillus* are common types of microorganisms used as probiotics, which can change the composition and formation of biofilms by inhibiting the growth of cariogenic bacteria such as *S. mutans* and *S. sobrinus*. Probiotics may play a direct or indirect role in the prevention of caries. They can directly interfere with the formation of biofilms and compete with oral microorganisms for nutrients and biochemicals to inhibit damage to the oral microenvironment. They also function by indirectly removing harmful microorganisms and stabilizing the normal state. Dairy products, an ideal carrier for probiotic management, can neutralize the acidic conditions in the mouth, inhibit the growth of cariogenic bacteria, inhibit the demineralization process, and promote enamel re-mineralization (Srivastava et al., 2016). One of the new methods being used therapeutically effects is whole bacterial replacement therapy, a method of fighting infection by replacing pathogenic microorganisms with harmless bacteria (Caglar et al., 2005). Probiotics play a significant role in preventing the development of dental caries. Probiotics help stimulate health-promoting microbiota while inhibiting pathological colonization and disease. They can prevent dental caries through mechanisms that buffer saliva pH, production of bacteriocins and enzymes (dextranase, mutanase, and urease), and competition for tooth surface adhesion and colonization to inhibit cariogenic bacteria and commensal microorganisms in the mouth (Sivamaruthi et al., 2020).

However, at the same time, probiotics also have some limitations in preventing dental caries, e.g., some probiotics such as *Lactobacillus* and *Bifidobacterium* in excess might increase the risk of caries (Takahashi and Nyvad, 2011; Zheng et al., 2019). Probiotics also pose a risk of infection in some populations, such as those with immune deficiencies. It may lead to increased likelihood of bacteremia and fungemia. Overuse of probiotics may lead to an increase in candida infections in oral or *Clostridium difficile*-associated diarrhea (Goldenberg et al., 2017). In the future, researchers should focus on isolating strains that are effective and safe for caries, and determine the corresponding dose, treatment time, and appropriate population through animal experiments and clinical trials (Table 2).

**TABLE 2:** Dental caries prevention measures.

Prevention strategy	Representative drug	Molecular mechanism	Advantages	Disadvantages	References
Ecological strategy	Nanoparticles	Interfere with the metabolism of cariogenic bacteria, inhibit biofilm formation and reduce demineralization of dental hard tissues	Broad-spectrum antimicrobial properties, good stability	Relatively expensive	Zhang et al., 2022
	Probiotics	Produces ammonia, prevents the colonization of cariogenic bacteria, maintains internal pH homeostasis, and interferes with biofilm formation	Common and relatively inexpensive	Excessive use may lead to the risk of infection	Reddy et al., 2021
	Dental caries vaccine	Triggers an antibody response in the nasal cavity, prompting the body to develop active immunity for preventive purposes	Long-lasting effect	Research and development difficulties	Chen et al., 2013
Traditional methods	Aminoglycosides	Binds to the A site on the 16S ribosomal RNA of the 30S ribosome to inhibit protein synthesis	Good efficiency and broad antimicrobial spectrum	Malabsorption through the gastrointestinal (GI) tract, administration by intravenous or intramuscular routes; More toxic, easy to cause disease nephrotoxicity, ototoxicity, and neuromuscular blockade disease; Biofilm promotion	Jana and Deb, 2006; Krause et al., 2016
	Penicillin	Inhibition of bacterial cell wall synthesis	Highly effective, low toxicity, and relatively inexpensive	Prone to allergic reactions	
	Quinolones	Interacts with DNA gyrase to inhibit bacterial DNA synthesis	Oral absorbability with good distribution in tissues and excellent interstitial fluid levels	Gastrointestinal disorders, central nervous system toxicity and rash and other adverse effects	
	Enolase	ENO1 is involved in glycolysis and is responsible for catalyzing the conversion of 2-PGA to PEP, and thus to pyruvate	Participates in a variety of biological processes, such as cell wall formation and RNA turnover, and as a fibrinogen receptor, with a wide distribution		Krucinska et al., 2019; Qiao et al., 2021
	Phages	Lysine can eliminate bacterial cell walls	Kill 95% of actinomycete bacteria	No removal of biofilm matrix	Szafrański et al., 2017b
	KSL	KSL down-regulates the growth and transformation of Candida albicans and inhibits the formation of biofilm	No specific target, antibacterial effect on drug-resistant bacteria, and less likely to lead to new drug resistance	Low efficacy against cariogenic bacteria, KSL does not have the typical complete amphiphilic structural features, and it is difficult to spontaneously embed into the cell membrane to form transmembrane ion channels	Theberge et al., 2013
	GH12	Inhibits the virulence factor of Streptococcus pyogenes, reduces EPS synthesis and inhibits biofilm synthesis, GH12 can reduce the number of Streptococcus pyogenes within the caries-causing three biofilms and destroy their integrity, thus making the biofilms easy to remove	Good stability, no significant cytotoxicity, Stabilizes helical structure and enhances the anti-microbial activity	Currently still in *in vitro* testing	Li X. W. et al., 2021
	Membrane active antibacterial molecules	SnF2 and AmF can inhibit lactate dehydrogenase enzyme(LDH) activity and inhibit the growth of Candida albicans	High efficacy, good drug resistance, broad- spectrum, selective for bacteria	Poor liveliness	
	Phenolic substance	Inhibit bacterial attachment and alter biofilm formation by altering cell surface properties and blocking protein activity, as well as altering the structure of bacterial-surface interactions	Oral, relatively common in life		Wang et al., 2013

### Caries vaccines proactively reduces the incidence of caries

Dental caries vaccines are mainly classified as subunit vaccines, mucosal vaccines, and DNA vaccines. Research on subunit vaccines has been carried out to include the functional part of the genome responsible for the production of Ag I/II or gtf or GBP (Patel, 2020). For example, the induction and enhancement of specific anti-carious salivary S-IgA antibodies by developing nasal sprays or nasal drops against caries allows intranasal immunization with safe and effective mucosal adjuvants to obtain protective immunity in the oral mucosa (Yan, 2013). S-IgA can also reduce *S. mutans* biofilm formation through early and late *S. mutans* HA disc adhesion effects. For vaccines to cross various hurdles, nanoparticle systems have been developed by incorporating anionic liposomes (AL) into chitosan/DNA (CS/DNA) complexes. By enhancing cellular uptake, the constructed AL/CS/DNA nanoparticles could deliver the anti-caries DNA vaccine pGJA-P/VAX into the nasal mucosa to enhance cellular uptake (Chen et al., 2013), which is conducive to the development of intranasal immunity, induces a specific salivary IgA antibody response, and reduces the enamel and dentin damage after *S. mutans* infection. Nasal immunization with KF-rPAc [in which *S. mutans* rPAc is fused directly to the C-terminus of *Escherichia Coli-derived* flagellin (KF)] can promote PAc-specific systemic and mucosal antibody responses, thus it can be used as a therapeutic mucosal vaccine against dental caries (Bao et al., 2015). The second generation flagellin-rPAc (KFD2-rPAc) was subsequently produced by replacing the main antigenic regions D2 and D3 of KF with rPAc, with low side effects and high anti-caries protection efficiency, which has lower Toll-like receptor 5 (TLR5) agonist efficacy and induces a lower systemic inflammatory response compared with KF-rPAc. Its fewer side effects and high protective efficiency make fusion protein KFD2-rPAc a promising candidate vaccine for dental caries. Various antibodies are also used for prevention (Yang et al., 2017). For example, mouse monoclonal antibodies, transgenic plants, egg yolk and milk are used to initiate local passive immunity against *S. mutans*. Passive immunity can control dental caries more safely than active immunity. This type of DNA vaccine has become a trend in caries vaccine research because of its high safety, stable antigenic protein expression and antigenicity (Patel, 2020). For future research, researchers should search for new target virulence genes or antigenic proteins, develop vaccines, use the best historical adjuvant and delivery technologies, and further enhance them with using nanotechnology, optimize caries scoring, and evaluate vaccine efficacy.

### Antimicrobial peptides (amps) applied to inhibit biofilm formation

In the process of anti-caries research, researchers have developed many different types of antimicrobial peptides to inhibit biofilm formation. The antimicrobial peptide GH12 has good stability and antibacterial performance, has no obvious cytotoxicity, inhibits the virulence factor of *S. mutans*, reduces EPS synthesis, and inhibits biofilm synthesis. In addition, by inhibiting the growth of S, *mutans* and enhancing the ecological competitiveness of *S. sanguinis* and *S. gordonii*, GH12 directly inhibits the occurrence of dental caries and the composition of multispecies biofilms (Jiang et al., 2018). KSL peptides have antibacterial activities against a variety of oral bacteria and fungi. They act via a mechanism similar to the inhibition of the formation of *S. mutans* biofilms and can reduce the viability of biofilm cells. The formation of dental plaque biofilm was inhibited by grafting serine diphosphate (-Ser(p)-Ser(P)-) into the interior structure of a bacterial peptide (Zhang et al., 2019). Among the novel AMP cyclic bacteriocins and their derivatives, Bac8c has a stronger antibacterial activity than cyclic and linear bacteriocins, and has the ability to reduce cell viability in biofilms formed in BioFlux systems (Ding et al., 2014). The specifically targeted antimicrobial peptide (STAMP) is a synthetic fusion peptide consisting of the selective “targeting domain” of *S. mutans* (C16) derived from CSP and the “killing domain” of the broad-spectrum antimicrobial peptide G2 (Philip et al., 2018). Researchers studied the permeability of biofilms to anions and cations to prevent and treat dental caries. For example, Chrysophsin-1 is a cationic antimicrobial peptide with broad spectrum bactericidal activity against gram-positive and gram-negative bacteria (Wang et al., 2012). pHly-1, a pH- and lipid-dependent AMP in conformational transitions, is capable of killing the acidic oral pathogen *S. mutans* under acidic conditions more effectively than chlorhexidine (CHX) (the gold standard for oral antimicrobial therapy) (Zhang et al., 2022).

### Anti-caries small molecules for the development of anti-plaque biofilm drugs

Small molecule compounds have the advantages of high cell permeability, low cost, and easy synthesis. In recent years, many small molecule synthetic compounds and natural products acting on various targets have become hot spots for the development of anti-plaque biofilm drugs.

SnF 2 and AmF might be options to treat existing biofilms and inhibit the production of new ones (Ayoub et al., 2022). Another method is to raise the pH in the oral environment using particular ingredients in toothpaste, which can also play a role in preventing caries. Supplementation with 1.5% arginine in a defined multispecies biofilm composed of *Streptococcus* promoted the proliferation of arginine deiminase system (ADS) negative bacteria through a rise of environmental pH (Berto et al., 2019) and positive *Streptococcus* in the ADS. In contrast, the incidence of caries *in vivo* was inversely correlated with ADS activity in saliva and dental plaque.

The first small molecule inhibitor to inhibit the formation of *S. mutans* biofilms was 2-amino-imidazole/triazole conjugate (2-AI/T) from marine natural products (Pan et al., 2015). It has been shown that lysine, the 29th amino acid in lactoferrin, one of the numerous multifunctional proteins found in or on all mucosal surfaces throughout the body, has been demonstrated to kill *S. mutans* and related acid-producing microorganisms and reduces proximal putrefaction (Fine, 2015). Substances such as polyphenols in tea components might inhibit the underlying mechanism of *S. mutans* attachment and subsequent biofilm formation on tooth surfaces by altering cell surface properties and blocking the activity of proteins and structures used by bacteria to interact with surfaces (Wang et al., 2013), and by changing the cell surface hydrophobicity to modulate streptococcal biofilm formation (Wang et al., 2021). Polyphenols, such as pentamers, trigonelline, caffeine, and chlorogenic acid in cocoa and coffee, have antibacterial effects, which can interfere with the adsorption of *S. mutans* to hydroxyapatite beads in salivary coatings. Ethyl gallate (EG) in grape seeds inhibited the formation of *S. mutans* biofilms in a dose-dependent manner (Gabe et al., 2019).

However, most of the small molecule compounds with anti-biofilm activity have unclear mechanisms of action and unknown *in vivo* pharmacokinetic parameters, which might affect bacterial growth prone to induce drug resistance.

### Other antibacterial agents

As an important antibacterial agent in the oral cavity, hydrogen peroxide is closely related to the release of extracellular DNA (eDNA) and the development of competent cells (Keke et al., 2017). The effect of iron oxide nanase or iron sulfide nanase greatly reduces the biofilm matrix of *S. mutans* in the presence of hydrogen peroxide-producing *S. gordonii*, which kills most of the bacteria through co-therapy, and inhibits living cells from forming a biofilm (Wang et al., 2020). However, in the lactoperoxidase system (LPO system), hydrogen peroxide is also involved in many enzymatic reactions, including the production of oxygen by catalase. Hypothiocyanates produced by the LPO system have antibacterial effects on cariogenic bacteria and block anaerobic bacteria associated with periodontal diseases, such as *Porphyroporphyria gingivalis* (Adams et al., 2017). The formation of *S. mutans* biofilms was inhibited by the synergistic treatment of Ag/ZnO nanocomposites with light-emitting diode (LED) radiation to exert a similar anti-caries function (Jiang et al., 2021). Arginine can be metabolized by arginine-utilizing oral species (e.g., *S. gordonii* and *Actinomyces spp.*) to produce a base, which counteracts the biofilm acidification process, regulates pH homeostasis within the oral biofilm, prevents overgrowth of acid-producing-acidic bacteria, and enhances the anti-caries activity of oral care products (Lamont et al., 2018). Mutanolysin can digest the cell wall of *S. mutans* BHT, thus reducing the attachment of *S. mutans*. Chlorhexidine (CHX) is the most commonly used antibacterial agent in oral care products. It is characterized by a broad spectrum and long-acting antibacterial activity, which can reduce a variety of bacteria associated with caries, such as *S. mutans* (Bacali et al., 2022). Adhesin subtype SpaP B is rich in amino acids, consistent with the formation of dental caries, and is the adhesin subtype produced by *S. mutans* with increased acid resistance. These subtypes match the individual differences in dental caries occurrence and can predict individual differences, which provides a basis for personalized oral care.

## Future prospects

The high recurrence rate and difficulty in prevention and treatment of caries has resulted in it becoming a serious public health problem that has been listed by the World Health Organization (WHO) as the third most important disease for prevention and treatment after cancer and cardiovascular diseases. Dental caries is very common in adults and children. Childhood caries (ECC) refers to caries in children under 6 years of age, which is often caused by night feeding (Mathur and Dhillon, 2018), frequent sugar consumption, lack of brushing, and enamel underdevelopment. The occurrence of dental caries in children has a great influence on their physical and mental health. Therefore, under the influence of bad living habits, we should also place greater emphasis on the impact of diet on dental caries. Studies have shown that the way in which probiotics are consumed may also affect the effectiveness of caries control, and that oral probiotics, whether in capsule or liquid form, increase *Lactobacillus* but do not affect *S. mutans* levels (Montalto et al., 2004). We should also consider whether the probiotic is tolerant of the oral environment, the maintenance effect and the biological activity of the drug in the oral cavity, etc. Therefore, further research is needed on how to achieve more efficient results in the prevention and treatment process. In the future, further development and research might be required to realize the use of a daily diet to prevent and treat dental caries. At present, there is no radical treatment for caries, and the pathogenesis of caries varies. It is necessary to further improve the detection rate of the factors that might lead to caries, improve the prevention effect, and facilitate the implementation of personalized care, thereby reducing the occurrence of caries and associated systemic diseases.

In the study of microecology from the macroscopic perspective, macro genomics technology has been introduced in oral microecology to overcome the limitation of microorganism pure culture by traditional isolation and culture methods, and to explore the diversity of oral microorganism microbiota, the functional activity, and the overall survival state of microorganisms. Based on the concept of metagenomics, high-throughput sequencing technology can be used to analyze the diversity of oral microbiota in caries and healthy children. On this basis, the structural changes of the microbiota in dental plaque at different stages of caries development and the related microbiota in the process from caries-free, caries leukoplakia, to caries cavity formation can be further studied. With the in-depth study of dental caries microecology, Pacbio real-time sequencing and Illumina Hiseq2000 metagenomic sequencing technology have been used to accurately locate the structural changes of dental caries microecology at the species level and find the enriched functional genes in patients with caries and their mechanisms of action, demonstrating that it is important to study the oral microecology of caries at the level of functional genes.

## Conclusion

Increasing our understanding of the factors affecting oral caries revealed that oral cariogenic microorganisms can increase the incidence of caries singly or via symbiosis. In addition, there is a close relationship among the occurrence of dental caries, the population effect, and the ecological theory of oral microorganisms. We further demonstrated that the mechanism of caries occurs through the interaction among the population effect promoting biofilm formation, the caries ecological hypothesis, and correlated genetic factors. With the rapid growth of science and technology, research has identified many oral cariogenic bacteria, from the traditional main cariogenic *S. mutans* to the newly discovered symbiotic bacteria and fungi such as *S.sobrinus*, *S.sanguinis*, and *Candida albicans*. By detecting the abundance and protein characteristics of various bacteria in the oral cavity, we can predict the trends in dental caries activity and take corresponding preventive measures to reduce the occurrence of dental caries. The combination of prophylactic measures from traditional antimicrobial peptides, probiotics based on the “ecological plaque hypothesis”, and ecological development will greatly reduce the potential risk of caries.

## Author contributions

YZ: writing – original draft, figures preparation, and review revision. SZ: review revision. JL: figures preparation and review revision. XL and YY: writing – original draft. JY: literature search. QW: conceptualization and writing – review and editing. YW, KC, and SD: review revision and touch up the article. All authors contributed extensively to the work presented in this article.
