# Corrigendum: Association of polymicrobial interactions with dental caries development and prevention

**DOI:** 10.3389/fmicb.2023.1237596

**Published:** 2023-06-20

**Authors:** Yimei Zhu, Ying Wang, Shuyang Zhang, Jiaxuan Li, Xin Li, Yuanyuan Ying, Jinna Yuan, Keda Chen, Shuli Deng, Qingjing Wang

**Affiliations:** ^1^Shulan International Medical College, Zhejiang Shuren University, Hangzhou, Zhejiang, China; ^2^Stomatology Hospital, School of Stomatology, Zhejiang University School of Medicine, Zhejiang Provincial Clinical Research Center for Oral Diseases, Key Laboratory of Oral Biomedical Research of Zhejiang Province, Cancer Center of Zhejiang University, Hangzhou, China

**Keywords:** cariogenic factors, microorganisms, quorum sensing, ecological perspectives, anti-caries, *Streptococcus* spp.

In the published article, the corresponding authors were Keda Chen^1*^, Shuli Deng^2*^, and Qingjing Wang^1*^. The correct corresponding author is only Qingjing Wang^1*^. The updated author list is as follows “Yimei Zhu^1†^, Ying Wang^2†^, Shuyang Zhang^1,2^, Jiaxuan Li^1^, Xin Li^1^, Yuanyuan Ying^1^, Jinna Yuan^1^, Keda Chen^1^, Shuli Deng^2^ and Qingjing Wang^1*^”.

The authors apologize for this error and state that this does not change the scientific conclusions of the article in any way. The original article has been updated.

